# Highly branched amylopectin binder for sulfur cathodes with enhanced performance and longevity

**DOI:** 10.1002/EXP.20210131

**Published:** 2022-01-24

**Authors:** Luke Hencz, Hao Chen, Zhenzhen Wu, Shangshu Qian, Su Chen, Xingxing Gu, Xianhu Liu, Cheng Yan, Shanqing Zhang

**Affiliations:** ^1^ Centre for Clean Environment and Energy, School of Environment and Science Griffith University, Gold Coast Campus Southport Queensland Australia; ^2^ School of Mechanical, Medical and Process Engineering Queensland University of Technology (QUT) Brisbane Queensland Australia; ^3^ School of Environment and Resources Chongqing Technology and Business University Chongqing P. R. China; ^4^ Key Laboratory of Materials Processing and Mold, Zhengzhou University Ministry of Education Zhengzhou P. R. China

**Keywords:** binders, highly branched amylopectin, lithium‐sulfur battery, lowly branched polysaccharides

## Abstract

The sulfur cathode of lithium‐sulfur (Li‐S) batteries suffers from inherent problems of insufficient mechanical strength and the dissolution of sulfur and polysulfides. Inspired by the extraordinarily resilient and strong binding force of the Great Wall binder, that is, the sticky rice mortar, we extracted highly branched amylopectin (HBA), the effective ingredient, as a low‐cost, nontoxic and environmentally benign aqueous binder for the sulfur cathode. The HBA‐based cells outperform the Li‐S batteries based on the traditional polyvinyldene diflouride (PVDF) binder and a lowly branched polysaccharide binder. The improved electrochemical performance in the HBA‐based cell could be attributed to two mechanisms. First, the branched structure of the HBA provides enhanced mechanical and adhesive properties, which allow for a robust electronic and ionic conductive framework to be maintained throughout the cathode after extended cycling. Second, the HBA shows enhanced polysulfide retention due to the polymer's abundant lone‐pair rich hydroxyl groups and the formation of C─S bonds between the HBA and polysulfides prohibits the shuttle effect of polysulfides. The improved mechanical properties and polysulfide retention function of the HBA binder facilitate the HBA‐based Li‐S battery to deliver a long cycle life of 500 cycles at 2 C while only displaying a capacity fading of 0.104% per cycle.

## Graphical abstract

Inspired by the extraordinarily resilient and strong binding force of the Great Wall binder, that is, the sticky rice mortar, we extracted highly branched amylopectin, the effective ingredient, as a low‐cost, nontoxic, and environmentally benign aqueous binder for high performance and long life sulfur cathode.

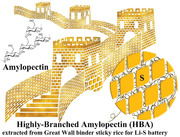

## 1 INTRODUCTION

As continual global population and economic growth increase society's demand for energy, the environmental consequences of energy production become more apparent.^[^ ^1^ ^]^ Since energy production is one of the major driving forces of carbon emissions and climate change^[^ ^2^ ^]^ and the majority of energy stems from nonrenewable resources,^[^ ^3^ ^]^ the requirement for clean and renewable energy is more urgent than ever.^[^ ^4^ ^]^ Wind‐ and solar‐based renewable energy have made significant inroads into energy grids across the world,^[^ ^5^ ^]^ but their implementation is hindered by their variable power supply,^[^ ^6^ ^]^ reaching up to 20% variability for wind energy.^[^ ^7^ ^]^ Therefore, energy storage systems such as rechargeable batteries are being implemented to supplement renewable energy generators.^[^ ^8^, ^9^ ^]^

Rechargeable batteries are also gaining traction in electric vehicles, which benefit from reducing CO_2_ emissions produced by internal combustion engines.^[^ ^10^ ^]^ However, rechargeable batteries are limited by their cost, energy density, and toxic components.^[^ ^11^ ^]^ As a result, novel battery chemistries are being investigated so that these shortfalls can be avoided. Lithium‐sulfur (Li‐S) batteries are promising next‐generation batteries that possesses a greater energy density than state‐of‐the‐art lithium‐ion batteries (LIBs), without the use of toxic transition metals in the cathode.^[^ ^12^ ^]^ However, they are limited in their commercial application due to some intractable technical issues.

The single greatest challenge hindering the practical application of the Li‐S battery is the polysulfide shuttle phenomenon (or effect), which arises from the fact that the lithium polysulfide reaction intermediates formed during cycling are readily soluble in the ether‐based electrolytes used in Li‐S cells.^[^ ^13^, ^14^, ^15^, ^16^ ^]^ This dissolution of the active materials at the cathode forms a concentration gradient, which causes the soluble species to migrate toward the anode, where they can be further reduced and damage the anode.^[^ ^17^ ^]^ These reactions cause a severe loss in Coulombic efficiency and rapid capacity fading.^[^ ^18^ ^]^ Additionally, the insulating nature of elemental sulfur (S_8_) as well as the solid discharge product in the Li‐S cell (Li_2_S_2_/Li_2_S) causes poor active material utilization and sluggish reaction kinetics, resulting in insufficient discharge capacity and poor performance at high C rates, respectively.^[^ ^19^, ^20^ ^]^ The conversion from sulfur to Li_2_S is also accompanied by a ≈80% increase in volume, which causes damage to the electronic conducting network and electrode delamination from the current collector, again reducing the electrochemical performance in Li‐S cells.^[^ ^21^, ^22^ ^]^ Finally, the use of the metallic lithium anode can cause dendrite formation, which may pierce the separator and causes operational safety concerns.^[^ ^23^ ^]^

Researchers in the field have directed their attention to the cathode,^[^ ^24^, ^25^, ^26^ ^]^ anode,^[^ ^27^ ^]^ electrolyte,^[^ ^28^ ^]^ and separator^[^ ^29^ ^]^ of the Li‐S cell in order to improve the electrochemical performance. Of these, the cathode of the Li‐S cell has received particular attention through the development and successful application of multifunctional sulfur hosts.^[^ ^11^ ^]^ When appropriately designed, sulfur hosts in Li‐S batteries can simultaneously mitigate multiple challenges within the Li‐S cell. For example, sulfur hosts with immense void structures, such as hierarchical porous carbons,^[^ ^30^ ^]^ can provide the necessary volume to accommodate a sufficiently large sulfur loading in the cathode while also providing abundant channels for electrolyte penetration during cycling.^[^ ^31^ ^]^ Sulfur hosts with inherent electronic conductivity can improve active material utilization and facilitate rapid charging/discharging.^[^ ^32^ ^]^ Heteroatom,^[^ ^33^ ^]^ transition metal oxide,^[^ ^34^ ^]^ or transition metal sulfide‐doped^[^ ^35^ ^]^ sulfur hosts can provide soluble polysulfide anchoring points to reduce the shuttle effect and catalyze the electrochemical reaction, improving the discharge capacity, reducing the capacity fading, and improving the kinetics at high C rates. Well‐designed sulfur hosts can incorporate many of these features at once.

Although remarkably effective at alleviating the aforementioned challenges, sulfur hosts can do little in the way of maintaining the intimate contact between the components of the composite sulfur cathode.^[^ ^36^ ^]^ Thus, researchers have turned their attention to the binders in the Li‐S cathode so that the structural damage and electrode delamination can be addressed.^[^ ^37^ ^]^ By utilizing binders with superior adhesive properties, a robust cathode composite can be maintained throughout extended cycles.^[^ ^38^ ^]^ However, adhesion between electrode components is not the only role that a binder can play in a Li‐S cathode, as demonstrated through the use of multifunctional binders.^[^ ^39^ ^]^ These binders go beyond simply proving improved adhesive properties by filling some of the roles traditionally left to the sulfur host, including providing chemical polysulfide anchoring via their functional groups or improving the electronic conductivity of the composite cathode.^[^ ^40^ ^]^ Among these, bio‐derived binders, including those derived from cyclodextrin^[^ ^41^, ^42^ ^]^ and gelatin^[^ ^43^, ^44^, ^45^ ^]^ have been widely applied in Li‐S cells due to their inherent mechanical properties, cost, chemical functionality, and environmental benignity.^[^ ^46^, ^47^, ^48^ ^]^

**SCHEME 1:**
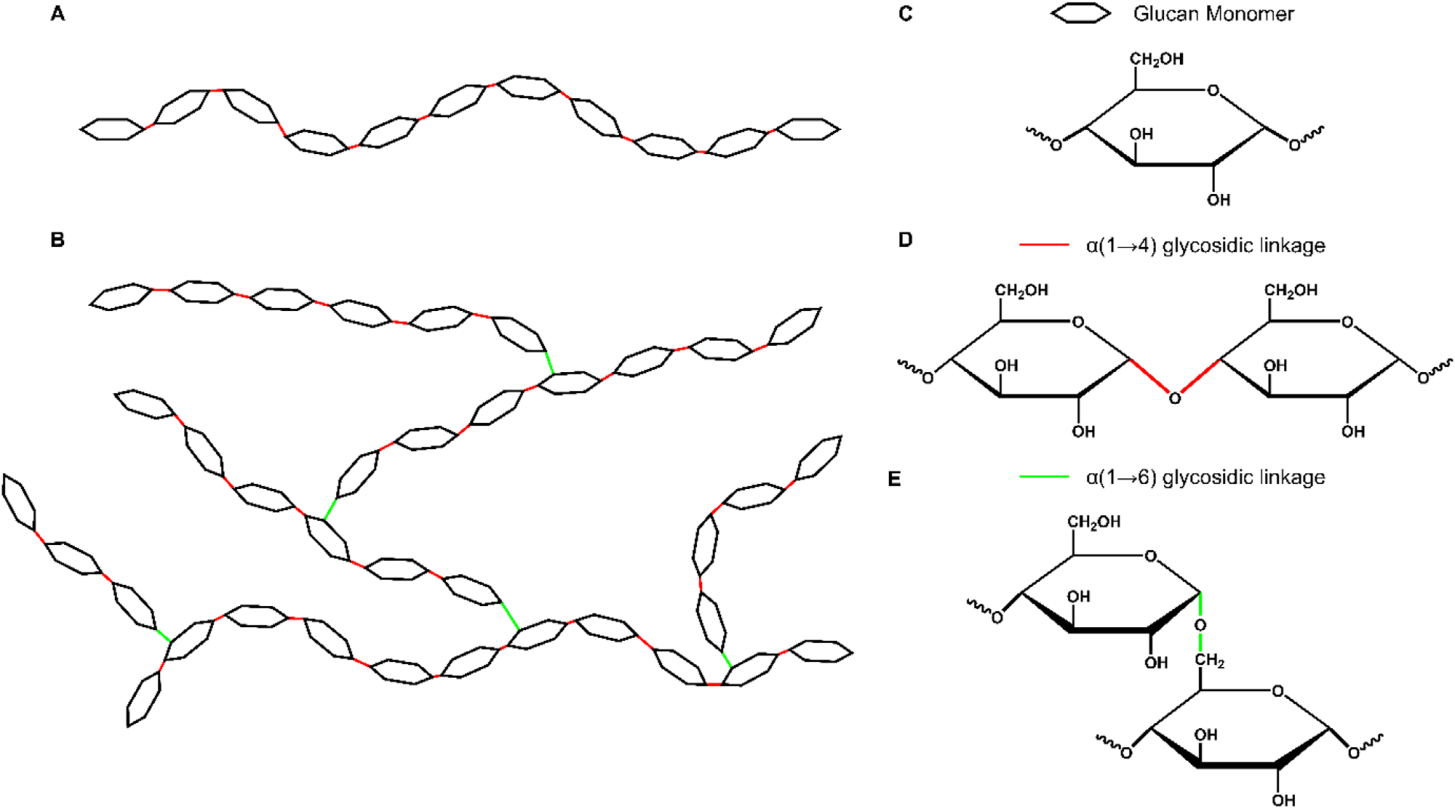
Representation of the (A) amylose and (B) amylopectin. (C) Chemical structure of the glucan monomer, α(1→4) glycosidic linkage (red, D), and α(1→6) glycosidic linkages (green, E)

Starch, mainly composed of the two polysaccharides, amylose (Scheme 1A) and amylopectin^[^ ^49^ ^]^ (Scheme 1B), is primarily used as food but has also found uses in textile, chemical production, paper binders, and adhesive applications.^[^ ^50^ ^]^ Both amylose and amylopectin consist of repeating glucose units (Scheme 1C) but differ in their structural configurations.^[^ ^51^ ^]^ Amylose is an essentially linear chain polymer, with α(1→4) glycosidic bonds (Scheme 1D) and a typical molecular weight around 10^6^ g·mol^−1^, whereas amylopectin displays both α(1→4) and α(1→6) linkages (Scheme 1E) resulting in a highly branched structure and an increased molecular weight (approx. 10^8^ g·mol^−1^).^[^ ^50^, ^52^, ^53^ ^]^ The different conformations of the two polysaccharides affect the resulting physical and chemical properties. Initially, starch is insoluble in water, but upon heating, amylose forms a nonviscous solution, whereas amylopectin remains insoluble and forms a viscous gel.^[^ ^54^ ^]^ The physical structure of the starch is also related to its digestibility, with starches ranging from rapidly digestible to resistant.^[^ ^55^ ^]^

Sticky (or glutinous) rice is a valuable starch source with favorable adhesive properties due to its high amylopectin content. Sticky rice has been used as a mortar ingredient in ancient China for over 3000 years^[^ ^56^ ^]^ and has been applied in constructing tombs, roads, cities, and even in the Great Wall of China.^[^ ^57^ ^]^ Starch has already been applied in LIBs with varying success^[^ ^58^, ^59^, ^60^, ^61^ ^]^ and is beginning to emerge in Li‐S applications.^[^ ^62^ ^]^ However, little attention has been paid to the highly branched amylopectin (HBA) polysaccharide found in starch. With its highly branched structure, the starch extracted from sticky rice is expected to deliver an improved electrochemical performance in Li‐S cells compared to previous works that have not considered the degree of branching in the starch.

This work applies HBA binder, which is near 100% amylopectin content, as a low‐cost and environmentally benign Li‐S cathode binder. For the control experiment, the lowly branched polysaccharide (LBP) binder is obtained from potatoes, which is composed of approximately 70% amylopectin and 30% amylose. As a result, the HBA binder displays improved mechanical properties and adhesion compared with the traditional Li‐S binder polyvinylidene difluoride (PVDF) and a mixed LBP binder. The HBA binder also displays the ability to chemically retard soluble lithium polysulfides due to its lone‐pair rich hydroxyl groups and C─S bond formation. These features allow the HBA‐based binder to deliver enhanced electrochemical performance in Li‐S cells compared to cells based on PVDF and LBP binders. The HBA‐based cell delivers a capacity fading as low as 0.104% over 500 cycles at a 2 C rate, superior rate kinetics, and better Li^+^ diffusion throughout the cathode. This improvement is particularly appealing as the HBA is derived from both an environmentally friendly source and extraction method, which could reduce the environmental impact of the Li‐S cell.

## 2 EXPERIMENTAL SECTION

### 2.1 Highly Branched amylopectin extraction

Sticky (glutinous) rice was obtained from the local Asian supermarket (Figure S1). The sticky rice was soaked in deionized (DI) water at room temperature for 24 h before refluxing at 80 °C for 4 h under magnetic stirring. The mixture was then cooled to RT and centrifuged. After centrifuging, the mixture contained a solid lower layer, a highly viscous gelatinous middle layer, and a low viscosity upper layer. The middle layer was separated, centrifuged again, and collected before being freeze‐dried. The collected extract is referred to as the HBA binder hereafter.

### 2.2 Materials characterization and mechanical characterization

The details of materials characterization and mechanical characterization can be found in Supporting Information.

### 2.3 Electrochemical characterization

For electrochemical testing, sulfur cathodes using PVDF, LBP, or HBA as a binder were fabricated with elemental sulfur, carbon black, and binder in a mass ratio of 60:30:10 and were denoted S/PVDF, S/LBP, and S/HBA, respectively. The electrode components were ground in a mortar and pestle before being made into an electrode slurry using NMP as the solvent. The slurry was cast using a gapped blade on carbon‐coated aluminum foil before being dried in a vacuum oven at 60 °C for 24 h and cut into disks with a diameter of 13 mm. The active material loading in the electrodes was approx. 0.5 mg·cm^−2^. Electrodes with a sulfur loading of ≈2 mg·cm^−2^ were also fabricated for high sulfur load testing. Electrodes fabricated from 80% carbon black and 20% HBA were also fabricated for electrolyte stability testing. Half‐cells were fabricated in an argon‐filled glovebox using either an S/PVDF, S/LBP, or S/HBA cathode, a lithium foil counter electrode, polypropylene (Celgard 2300) separator, and 1 M LiTFSI in DOL/DME (1:1, v/v) with 0.2 M LiNO_3_ as the electrolyte. The electrolyte/sulfur (E/S) ratio was kept constant at 1 mg:20 μl for all half‐cells. The half‐cells underwent galvanostatic charge/discharge testing on a Neware Battery Testing System (Neware, China) with a 1.7–2.7 V voltage window in an oven set at 30 °C. For the 0.5 C (1 C = 1672 mA·g^−1^) and high‐loading 0.2 C testing, an electrochemical sulfur infiltration pre cycle of 0.05 C was performed before cycling at the specified rate. For the 2 C testing, a 0.2 C pre cycle was utilized. Electrochemical impedance spectroscopy (EIS) was carried out on a Biologic SP‐200 (Biologic, France) with the AC set to 5 mV and a frequency range of 10 mHz to 100 kHz. Cyclic voltammetry (CV) testing was also carried out on the Biologic SP‐200.

## 3 RESULTS AND DISCUSSION

The extraction of sticky rice binder is outlined in Figure S1. After processing and centrifuging, the upper‐most low viscosity layer and the lower insoluble pellet were separated from the highly viscous central supernatant layer to obtain the HBA binder. The HBA was expected to have a high amylopectin content due to the relative viscosities of amylopectin and amylose solutions, which were exploited during processing.^[^ ^63^ ^]^ As a result, a simple separation is achieved by simply extracting the HBA in water, and a highly branched and environmentally benign binder is obtained. The obtained HBA and LBP binder were first characterized by TGA to qualitatively observe their thermostability (Figure S2A). Both samples experience a mass loss up to a temperature of about 150 °C, attributed to water incorporated into the starch matrix. The amount of branching in the starch can be estimated by observing the temperature at 50% mass loss, where a higher degree of branching gives a higher temperature at 50% mass loss.^[^ ^64^ ^]^ It can be inferred that the HBA sample has a higher degree of branching when compared to the LBP sample XRD analysis was also carried out to observe the crystal structure, with the diffraction patterns presented in Figure S2B. The PVDF sample displays diffraction peaks at 18.33, 19.89, and 26.23 ^o^, confirming the presence of α‐phase PVDF,^[^ ^65^ ^]^, which is nonpolar and semi‐crystalline.^[^ ^66^ ^]^ The diffraction pattern of the LBP displays both crystalline and amorphous regions, with the amylose fraction of the LBP responsible for the crystalline phase, while the amorphous phase can be attributed to the amylopectin within the LBP.^[^ ^67^ ^]^ In contrast, the HBA diffraction pattern is entirely amorphous, confirming the high amylopectin content suggested by the TGA.

**FIGURE 1:**
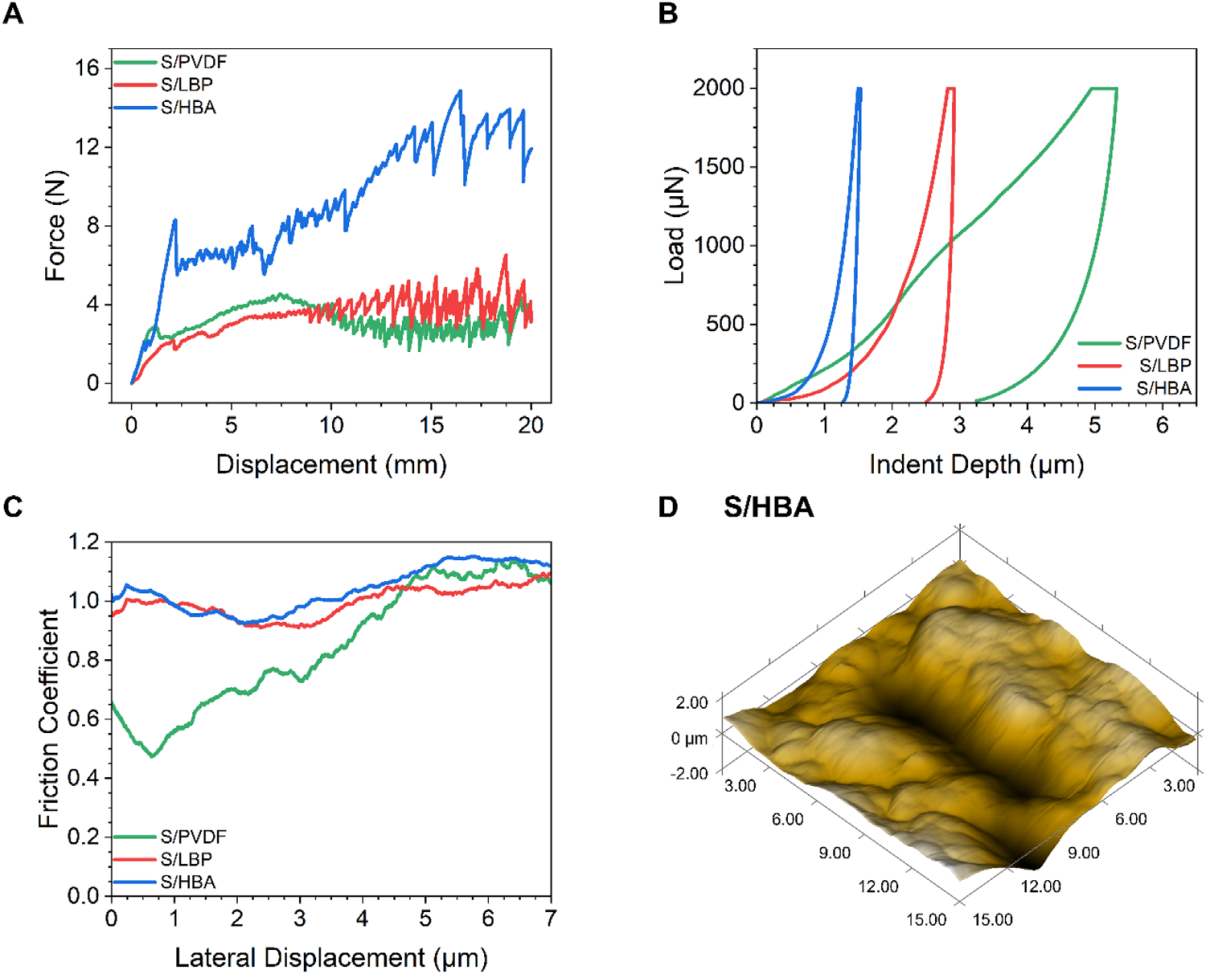
Mechanical test of the S/HBA electrode: (A) 90^o^ mechanical peel, (B) nano‐indentation tests, and (C) nano‐scratch tests. (D) Scanning probe microscopy (SPM) image of S/HBA

To investigate the mechanical and adhesive properties of the as‐prepared samples, 90^o^ peel‐off, nano‐scratch, and nano‐indentation testing were carried out on the S/PVDF, S/LBP, and S/HBA electrodes, with the results shown in Figure 1. The mechanical peel test results in Figure 1A show that the S/PVDF and S/LBP samples initially display a roughly equivalent resistance to peeling, with the S/LBP sample providing slightly better adhesion as the peel distance increases. In sharp contrast, the S/HBA sample displays a much larger initial resistance to the pulling force, shown at low displacement values, and a dramatically increased adhesion force toward the end of the peel test. These results clearly show that the S/HBA displays much better adhesion than both S/PVDF and the S/LBP samples.^[^ ^68^ ^]^ The hardness of the three electrodes was also evaluated by nano‐indentation testing. A shallower indentation at the same load suggests a more rigid material. The results in Figure 1B show that the indentation depth at a load of 2000 μN for the S/HBA, S/LBP, and S/PVDF samples was 1.53, 2.91, and 5.32 μm, respectively, which displays the rigid and robust nature of the S/HBA electrode.^[^ ^69^ ^]^ Nano‐scratch testing was also carried out to evaluate the adhesive properties and homogeneity of the S/PVDF, S/LBP, and S/HBA electrodes. As the results in Figure 1C show, both the S/HBA and S/LBP samples display smoother profiles than the S/PVDF sample, suggesting a better homogeneity of these electrodes. What is more, the average friction coefficients for the S/HBA, S/LBP, and S/PVDF samples are 1.04, 0.99, and 0.84, respectively. The higher friction coefficient in the nano‐scratch test suggests a higher adhesion in the electrodes.^[^ ^46^ ^]^. Additionally, the scanning probe microscopy (SPM) image from the S/HBA sample (Figure 1D) shows a more homogeneous scratch and dense electrode surface than the SPM images from the S/PVDF and S/LBP samples, shown in Figures S3A and S3B, respectively. Overall, the results in Figure 1 show that the S/HBA possesses better adhesion, more homogeneity, and increased hardness than the S/LBP and S/PVDF samples.

**FIGURE 2:**
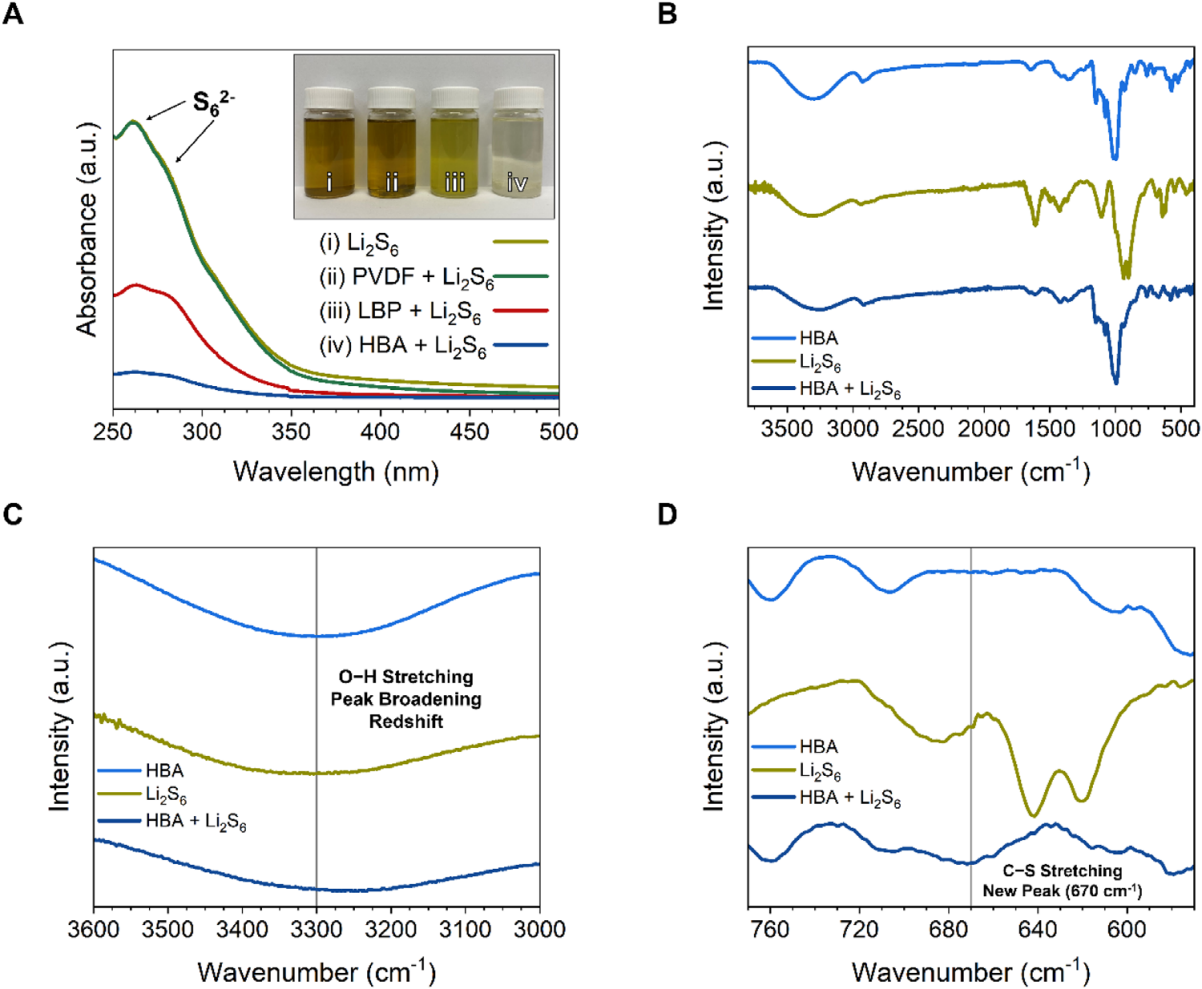
(A) UV–Vis spectra of the Li_2_S_6_, Li_2_S_6_ + PVDF, Li_2_S_6_ + LBP, and Li_2_S_6_ + HBA solutions with digital photographs of the respective solutions (inset). (B) FTIR spectra of the HBA, Li_2_S_6_, and HBA + Li_2_S_6_ samples. Magnified regions of the HBA, Li_2_S_6_, and HBA + Li_2_S_6_ FTIR spectra showing the (C) O─H stretching and (D) C─S stretching regions

The working mechanisms of HBA binder toward the soluble polysulfides in the electrolyte, which is significant in the shuttle effect, have been verified by the UV–Vis spectroscopy and FTIR spectra. The adsorption experiment was first conducted to demonstrate whether the HBA could retain soluble polysulfides. 100 mg of either the PVDF, LBP, or HBA samples were exposed to a 0.01 M solution of Li_2_S_6_ in DOL:DME for 4 h, after which the supernatant solution was studied using UV–Vis spectroscopy (Figure 2A). The spectra from the solution of Li_2_S_6_ contain a broad absorbance peak around 250 ‐ 280 nm, which is characteristic of the S_6_^2−^ anion.^[^ ^70^ ^]^ The spectra from the supernatant solutions clearly show that the PVDF has no interaction with the polysulfides as the peak height remains unchanged. Conversely, both the LBP and HBA display some ability to adsorb soluble polysulfides as the S_6_^2−^ peak height has reduced in both cases. As the HBA displays the smallest peak ascribed to S_6_^2−^, it is inferred that it possesses the best polysulfide anchoring ability. These results could visually be confirmed by the digital photographs attached in the inset of Figure 2A, which shows the Li_2_S_6_ solution that was exposed to the PVDF experienced no color fading, the solution exposed to the LBP showed minimal color fading to light yellow, and the solution exposed to the HBA sample experienced a significant color fading from dark brown to almost colorless. Then, Figure. 2B presents the FTIR spectra of the HBA, Li_2_S_6_, and HBA + Li_2_S_6_ samples with the FTIR spectra of the PVDF, Li_2_S_6_, and PVDF + Li_2_S_6_ samples and the LBP, Li_2_S_6_, and LBP + Li_2_S_6_ samples shown in Figures S4A and S4B, respectively. The magnified regions of the peaks associated with the O─H and C─S stretching in the HBA samples are provided in Figure 2C,D. The peak located at around 3300 cm^−1^ in the HBA sample is assigned to the O─H stretching vibration^[^ ^71^, ^72^ ^]^ (Figure 2C). After Li_2_S_6_ exposure, it can be observed that the O─H stretching vibration peak experiences a downshift from 3296 to 3233 cm^−1^ while also becoming broader. The downshift could signify a coordination interaction between the lone‐pair rich hydroxyl groups and the Li^+^ from Li_2_S_6_.^[^ ^73^ ^]^ The appearance of the new peak at 670 cm^−1^ (Figure 2D) can be assigned to the formation of the C─S bond after the HBA was exposed to Li_2_S_6_.^[^ ^46^, ^74^ ^]^ These two interactions suggest the possible mechanism of the polysulfide adsorption delivered by the HBA. When compared to the LBP (Figure S4B) sample, it can be observed that the O─H stretching peak only experiences a downshift of about 13 cm^−1^ and does not display a new peak associated with the C─S bond, which provides evidence as to why the LBP sample only displays mild interactions with the Li_2_S_6_ solution.

**FIGURE 3:**
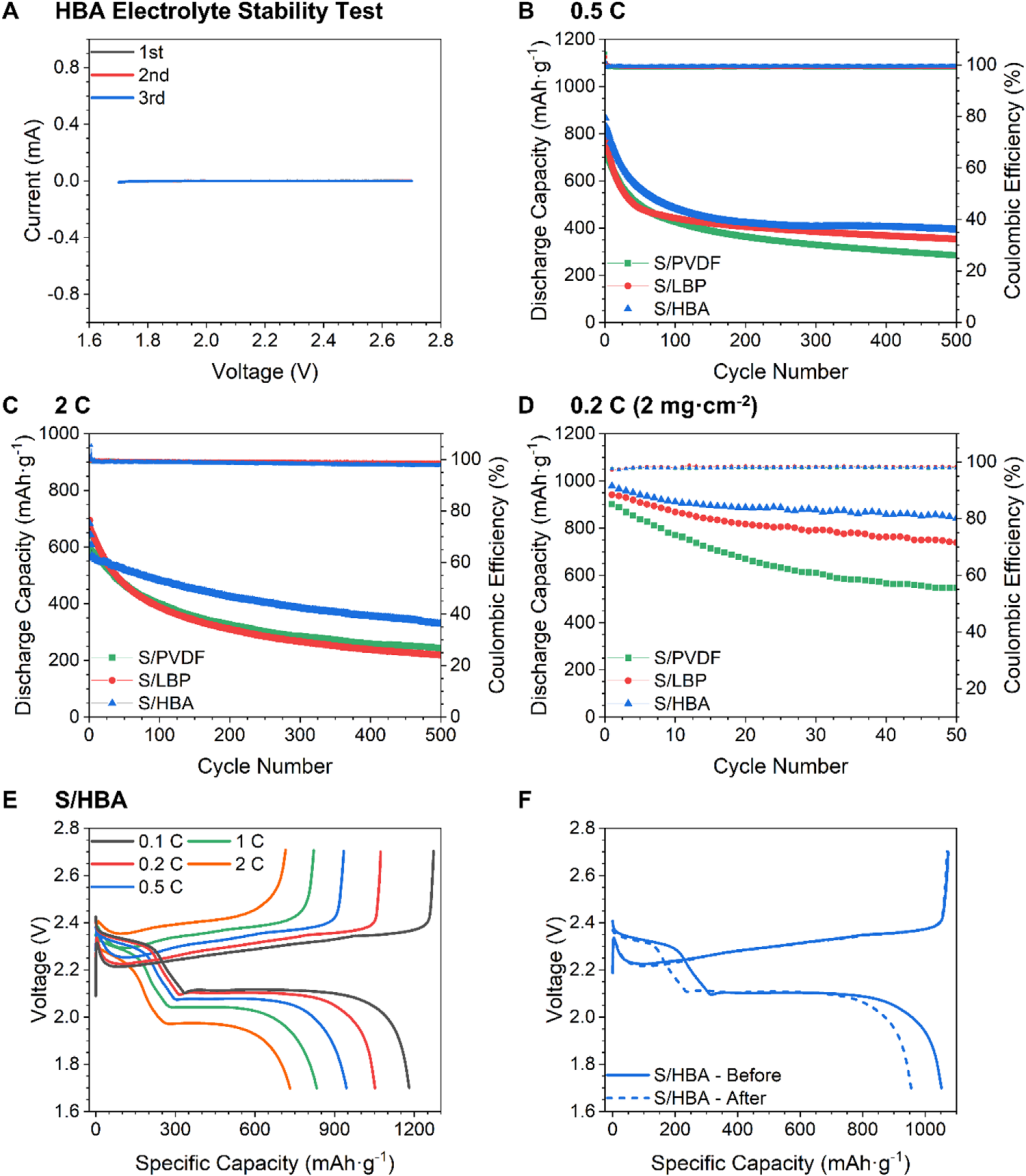
(A) Cyclic voltammogram of the CB:HBA (80:20) electrode for electrolyte stability testing. Cycle performance of the S/PVDF, S/LBP, and S/HBA electrodes at (B) 0.5 C, (C) 2 C, and (D) 0.2 C. Capacity/voltage profiles of the S/HBA cell (E) during rate capability testing and (F) for the two cycles at 0.2 C during and after rate capability testing

The electrochemical stability of HBA was tested based on electrodes consisting of 80% carbon black and 20% HBA which were assembled into half‐cells. These cells were then subjected to cyclic voltammetry at 0.05 mV·s^−1^ to investigate the stability of the HBA in the electrolyte, with the voltammogram shown in Figure 3A. As there is no electrochemical peak during the anodic or cathodic scans, it can be inferred that the HBA is stable in the electrolyte during charge/discharge and does not contribute electrochemically to the reaction. Electrochemical evaluation was carried out to determine if the polysulfide anchoring ability and superior mechanical properties of the HBA actualized an enhanced electrochemical performance. Sulfur cathodes using the HBA, LBP, and PVDF binders were used to fabricate sulfur composite electrodes and are denoted as S/HBA, S/LBP, and S/PVDF, respectively. The sulfur loading in the electrode is around 0.5 mg cm^−2^. First, the long cycling stability at different current densities was carried out on the S/PVDF, S/LBP, and S/HBA batteries through galvanostatic charge‐discharge test. Figure 3B shows the cells’ discharge capacity and Coulombic efficiency at 0.5 C (1 C = 1672 mA·g^−1^). The discharge capacity in the initial cycle for the S/PVDF, S/LBP, and S/HBA‐based cells are 751, 817, and 866 mAh·g^−1^, respectively. After 500 cycles, the S/PVDF, S/LBP, and S/HBA cells deliver a capacity of 285, 354, and 392 mAh·g^−1^, corresponding to a 0.124%, 0.113%, and 0.109% capacity fade per cycle, respectively. When the current density is increased to 2 C, the superior capacity retention of the S/HBA‐based cell is still highlighted in Figure 3C with the cells subjected to 500 charge/discharge cycles. In the initial cycles at 2 C, the S/HBA cells undergo a rapid capacity fading that can be attributed to an activation process, which is a common phenomenon in Li‐S batteries.^[^ ^75^ ^]^ Despite this, the S/HBA cell presents the smallest capacity fade from the 4th cycle onward and delivers the highest discharge capacity of 328 mAh∙g^−1^ at the 500th cycle. The capacity fade per cycle during testing is 0.124%, 0.137%, and 0.104% for the S/PVDF, S/LBP, and S/HBA cells, with the fade per cycle for the S/HBA cell dropping to 0.0845% when calculated from the fourth cycle onward. The amount of active materials at the cathode is a crucial parameter for Li‐S battery commercialization.^[^ ^36^ ^]^ Therefore, S/PVDF, S/LBP, and S/HBA electrodes were fabricated with a high sulfur loading of roughly 2 mg∙cm^−2^ and subjected to charge/discharge testing at 0.2 C, with the results shown in Figure 3D. Even at a higher material loading, the S/HBA electrode delivers a higher initial capacity of 978 mAh∙g^−1^, a reversible capacity of 842 mAhg^−1^ at the 50th cycle, and a capacity fading of 0.278% per cycle over the tested range, again outperforming both the S/LBP and S/PVDF batteries. Thus, it can be concluded that S/HBA batteries have higher reversible capacity, long cycle stability, and sulfur loading than batteries made with PVDF or LBP binders.

Rate performance is another critical parameter for electrochemical cells because charging/discharging in real situations is rarely galvanostatic. Thus, to evaluate the performance under differing current densities, rate performance testing was carried out with the results for the S/HBA cell shown in Figure 3E and the results for the S/PVDF and S/LBP cells shown in Figures S5A and S5C, respectively. The cells were charged and discharged at progressively larger current densities before being subjected to a final cycle at 0.2 C to observe the electrochemical performance after high rate testing, with the charge/discharge profile of the two 0.2 C cycles shown in Figure 3F and Figure S5B and S5D. The S/HBA cell delivers a higher discharge capacity and lower overpotential at all current densities than the S/LBP and S/PVDF cells, reflecting the galvanostatic charge/discharge results. Additionally, when observing the charge curve at 2 C, the S/HBA cell is smooth with no irregularities, whereas the same curve in the S/PVDF cell shows inhomogeneity. Furthermore, it can be observed that even at 2 C, the S/HBA cell exhibits the two clear characteristic discharge plateaus associated with the Li‐S battery, whereas the plateaus in both the S/PVDF and S/LBP curves become less pronounced, which may explain the enhanced performance of the S/HBA cell at higher current densities. Upon observing the charge/discharge profile of the 0.2 C cycle before and after rate testing, it is clear that the S/HBA cell delivers a higher initial discharge capacity and a higher discharge capacity after cycling at high rates. The discharge capacity retention after rate performance testing was found to be 86.3%, 81.7%, and 94.6% retention for the S/PVDF, S/LBP, and S/HBA‐based cells, respectively. The rate performance results demonstrate that the S/HBA cell delivers higher discharge capacities at high current densities and retains more capacity when reverting to lower current density cycling than the S/LBP and S/PVDF cells.

**FIGURE 4:**
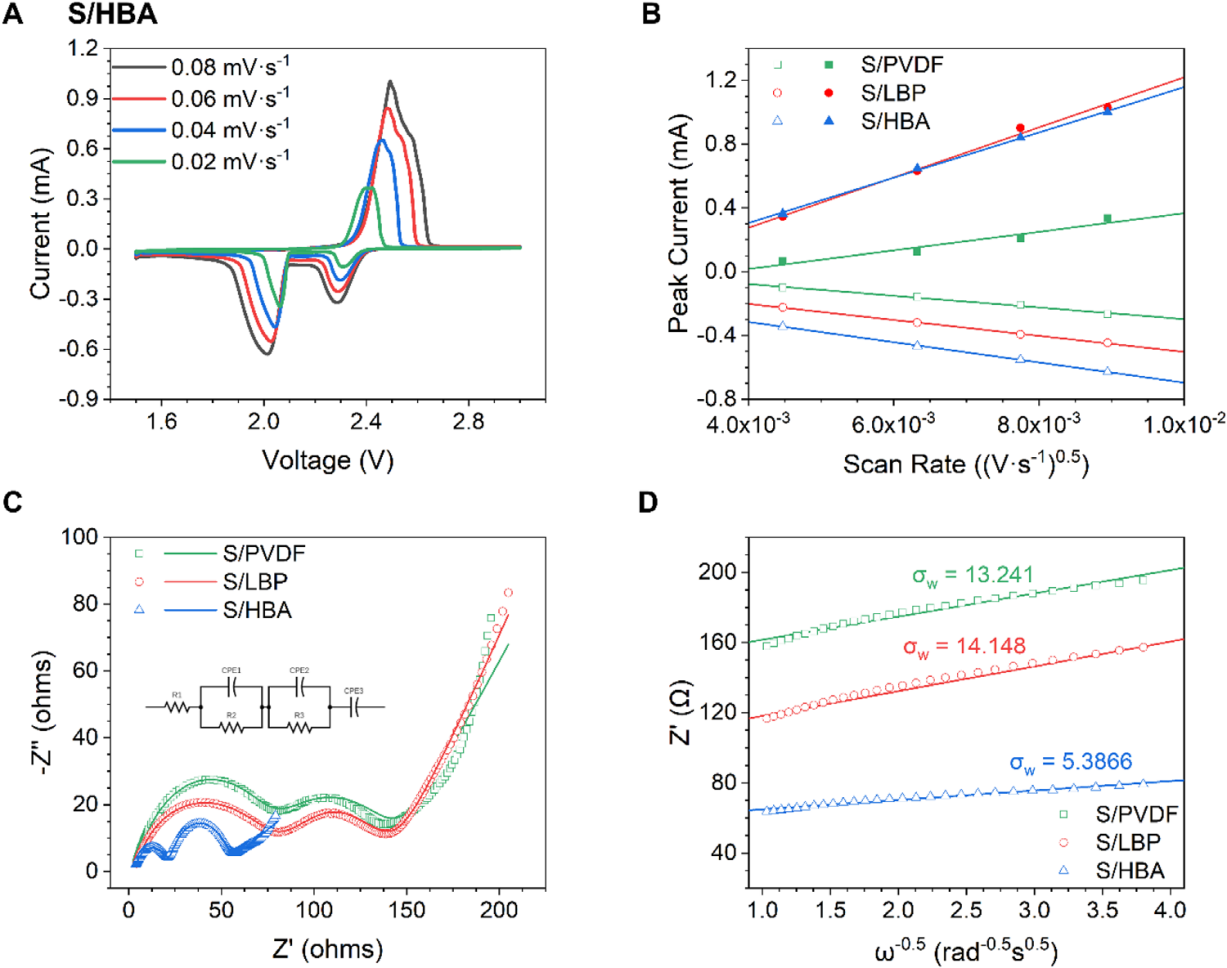
(A) Cyclic voltammograms (CV) of the S/HBA electrode. (B) Peak current (*I*_P_) versus the square root of the scan rate during CV testing. (C) EIS spectra of the S/PVDF, S/LBP, and S/HBA electrodes after 20 cycles at 0.2 C with the equivalent circuit using for EIS fitting (inset). (D) Zʹ’ versus *ω* ^−0.5^ showing the Warburg factor (*σ*_W_)

To investigate the reaction kinetics in the S/PVDF, S/LBP, and S/HBA cells, cyclic voltammetry at varying scan rates was carried out so that the lithium‐ion diffusion (*D*_Li_) properties could be evaluated (Figure 4 and Figures S6A and S6B, respectively).^[^ ^76^ ^]^ The cells display similar electrochemical reaction voltages; however, the S/HBA cell displays much higher peak currents at all reaction steps than the S/PVDF cell. Compared with the S/LBP cell, the S/HBA cell displays a similar peak current in the anodic scan but a much higher peak current in the cathodic scan, suggesting much faster reaction kinetics.

After plotting the peak current (*I*_P_) against the square of the scan rate (*v* ^0.5^) (Figure 4B), a linear relationship is observed, indicating a diffusion‐controlled process.^[^ ^76^ ^]^ Thus, according to the Randles–Sevcik equation (Equation 1),^[^ ^77^ ^]^ a steeper gradient in Figure 4B can be related to a higher *D*_Li_.^[^ ^78^ ^]^ As a result, the S/HBA cell displays better Li_+_ diffusion when compared to the S/PVDF and S/LBP cells. This phenomenon may explain the enhanced performance, especially at the higher current rate of 2 C.

(1) $$I_{P}=2.686\cdot 10^{5}n^{1.5}AD^{0.5}Cv^{0.5}$$

(2) $$\left|Z^{\prime }\right|=R_{\mathrm{ct}}+R_{\mathrm{int}}+\sigma_{W}\omega^{-0.5}$$

(3) $$D_{\mathrm{Li}}=\frac{R^{2}T^{2}}{2A^{2}n^{4}F^{4}C^{2}{\sigma_{W}}^{2}}$$

To further investigate the electrochemical performance, the S/PVDF, S/LBP, and S/HBA cells were also subjected to EIS analysis after 20 cycles at 0.2 C with the experimental and fitting results shown in Figure 4C. The equivalent circuits used for fitting are shown in the inset of Figure 4C. Depressed semi‐circles in the high‐ to medium‐frequency region of the spectra can be observed, which can be attributed to the electrolyte resistance (*R*_e_), charge transfer resistance (*R*_ct_), and interface/solid electrolyte interface resistance (*R*_int_), respectively.^[^ ^79^ ^]^ As the S/HBA cell displays lower resistances in all cases (Table 1), it can be inferred that the HBA better maintains the conductive carbon network after cycling compared to when PVDF or LBP is used as a binder. The linear portion of the curve in the low‐frequency region of Figure 4C can be attributed to the Warburg (*W*_O_) impedance in the cells.^[^ ^80^ ^]^ By graphing the real portion of the impedance (*Z*ʹ) in the linear region of the EIS spectra and *ω* ^−0.5^ (Figure 4D), the Warburg factor (*σ*_W_) can be calculated through the equation shown in Equation (2).^[^ ^81^ ^]^ The Warburg factor is inversely proportional to *D*_Li_, as shown in Equation (3).^[^ ^82^ ^]^ As the S/HBA cell displays the smallest *σ*_W_, it is inferred that the S/HBA cell displays the best lithium‐ion diffusion throughout the electrode matrix, again supporting the enhanced electrochemical performance at the high discharge rate of 2 C.

**TABLE 1:** The impedance calculated from EIS analysis

	S/PVDF [Ω]	S/LBP [Ω]	S/HBA [Ω]
*R*_e_	3.12	1.93	2.62
*R*_ct_	73.12	58.85	17.71
*R*_int_	63.72	37.71	27.51

**FIGURE 5:**
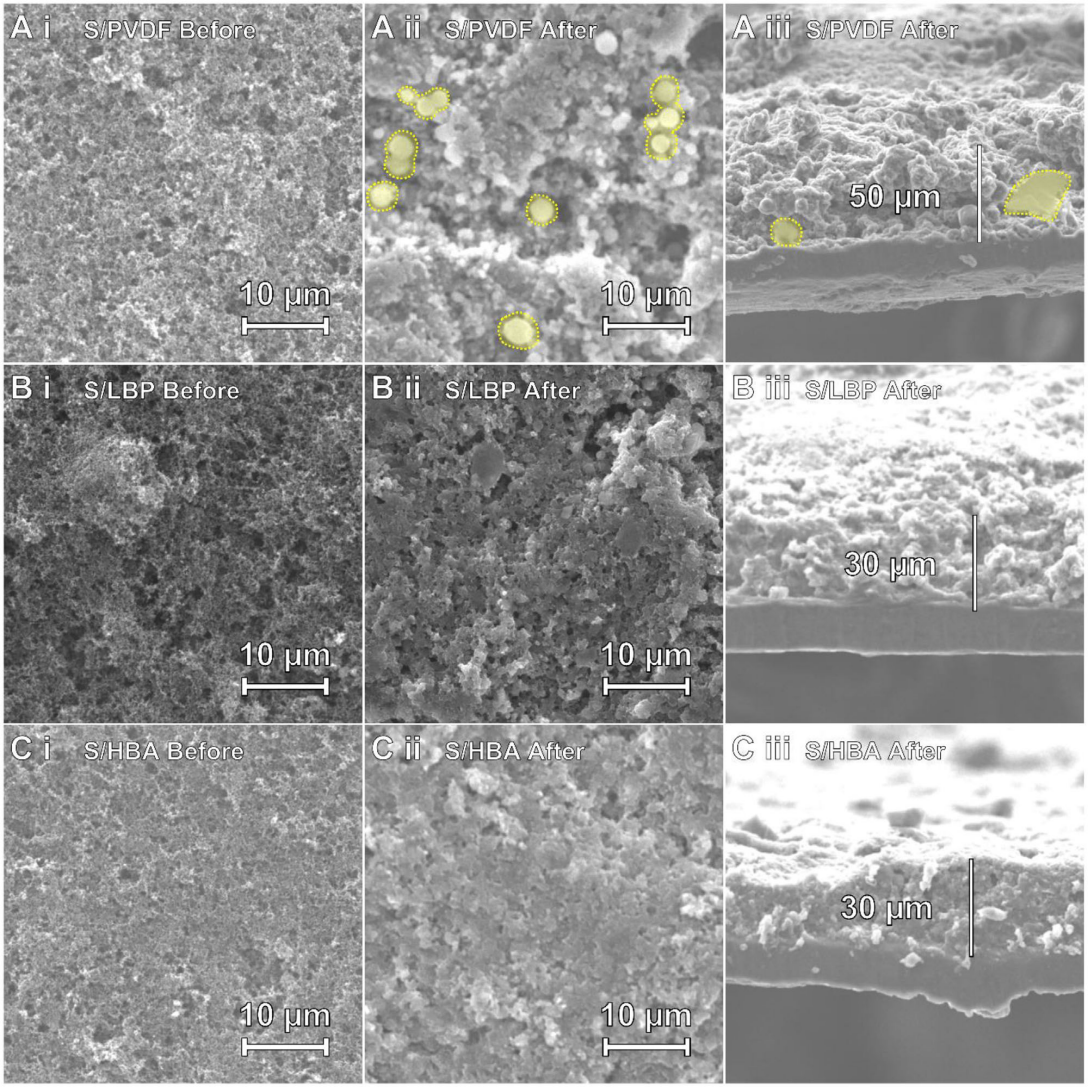
SEM images of the (A) S/PVDF, (B) S/LBP, and (C) S/HBA electrode's (i) surface before cycling, (ii) surface after cycling, and (iii) cross‐section after cycling

To further explain the adhesion ability of the HBA binder in S electrode, post‐mortem SEM and EDS analysis was carried out before and after the high‐loading S/HBA, S/LBP, and S/PVDF cells were subjected to 20 cycles at 0.2 C. Figure 5 shows the morphology of the S/PVDF (Figure 5A), S/LBP (Figure 5B), and S/HBA (Figure 5C) electrode's (i) surface before cycling, (ii) surface after cycling, and (iii) cross‐section after cycling. Before cycling, the S/PVDF, S/LBP, and S/HBA electrodes display relatively comparable morphologies. However, after cycling, the difference between the electrodes becomes pronounced. Figure 5C i shows that the S/HBA electrode maintains a relatively homogeneous surface with minimal large pits, with no observable Li_2_S/Li_2_S_2_ formation remaining on the electrode surface. The S/LBP electrode displays a similar morphology to the S/HBA electrode, albeit with slightly more surface irregularities (Figure 5Bii). In contrast, Figure 5Aii shows that after cycling, the S/PVDF electrode displays large pits and evident precipitation of the active materials (yellow). Cross‐sectional images of the high‐loading electrodes were also taken to observe the lateral homogeneity of the three electrodes. As shown in Figure 5Aiii, the S/PVDF electrode is roughly 50 μm thick and highlights that the extremely rough surface morphology persists throughout the whole depth of the electrode. The evident electroactive material precipitation is also highlighted in the cross‐sectional view. Conversely, the two starch‐based electrodes display a more compact cross‐section with depths of roughly 30 μm, as shown in Figure . The HBA‐based cross‐section displays the electrodes’ dense and homogeneous morphology, reflecting the surface morphology SEM images discussed previously and is attributed to the HBA binder's outstanding mechanical and adhesive properties.

To examine the elemental distributions and homogeneity of the electrodes, energy‐dispersive X‐ray spectroscopic (EDS) analysis was carried out on the cycled electrodes, with the results shown in Figure S7. The EDS results demonstrate that the S/HBA electrode was the most homogeneous after cycling, with the S/PVDF being the least. Overall, the smooth and homogenous electrode morphology, achieved through the enhanced mechanical and adhesive properties of the HBA, suggests the maintenance of a robust electronically conductive network. This feature, combined with the polysulfide anchoring ability of the HBA, can explain the enhanced capacity retention during charge/discharge testing and the enhanced reaction kinetics observed in the CV and EIS testing.

## 4 CONCLUSION

Natural polysaccharides binders, including HBA and LBP binders, were successfully extracted, characterized, and applied during the fabrication of sulfur cathodes (i.e., the S/HBA and S/LBP electrodes) for Li‐S batteries. The S/HBA cathode significantly outperforms the conventional S/ PVDF and S/LBP cathodes due to the highly branched structure and abundant hydroxyl functional groups of the HBA. After 500 cycles, the S/HBA cathode could still deliver a reversible capacity of 396 mAh∙g^−1^ (0.101% fading per cycle) at 0.5 C and 328 (0.104% fading per cycle) at 2 C, which is remarkable in comparison to both the S/LBP and S/PVDF cathodes. Such an enhanced performance of the S/HBA cells can be explained through two mechanisms. First, the HBA possesses the ability to chemically retain soluble polysulfide molecules at the cathode as it possesses abundant lone‐pair rich hydroxyl groups along with the ability to form the C─S bonds with soluble polysulfides. Second, HBA displays outstanding mechanical properties that can help maintain a robust conductive network after extended cycling, facilitating the rapid electrochemical reaction kinetics of the charge and discharge processes and evidenced by the EIS and CV characterizations. Furthermore, HBA could be readily obtained from the environment, representing a low‐cost, nontoxic, and sustainable natural product. Therefore, this work inspires the modern battery industry to develop toward high performance, low‐cost, and environmentally friendly trends and sustainability.

## CONFLICT OF INTEREST

Shanqing Zhang is a member of the *Exploration* editorial board. The authors declare no conflict of interest.

## AUTHOR CONTRIBUTIONS

Luke Hencz and Hao Chen: conceptualization of the idea, experimentation, manuscript preparation. Zhenzhen Wu, Shangshu Qian, and Su Chen: critical experiment, data analysis, and mechanical properties testing. Xingxing Gu, Xianhu Liu, Cheng Yan, and Shanqing Zhang: conceptualization of the idea, manuscript preparation, and funding.

## Supporting information

Supporting information
