# Supplementary material for: Highly branched amylopectin binder for sulfur cathodes with enhanced performance and longevity
Source: Exploration (Beijing). 2022 Jan 24;2(1):20210131. doi: 10.1002/EXP.20210131 (PMC10190977; doi:10.1002/EXP.20210131)
Supplement: Supplementary file 1 — Supporting information [file EXP2-2-20210131-s001.docx]

**Supporting Information:**

**Highly-Branched Amylopectin Binder for Sulfur Cathodes with Enhanced Performance and Longevity**

## Luke Hencz^a^, Hao Chen^a,b*^, Zhenzhen Wu^a^, Shangshu Qian^a^, Su Chen^b^, Xingxing Gu^a,c^, Xianhu Liu^d^, Cheng Yan^b^, Shanqing Zhang^a*^

a: Centre for Clean Environment and Energy, Griffith University Gold Coast Campus, Southport, QLD, 4222, Australia

b: School of Mechanical, Medical and Process Engineering, Queensland University of Technology (QUT), Brisbane, QLD, 4000, Australia

c: School of Environment and resources, Chongqing Technology and Business University, Chongqing, 400044, China

d: Key Laboratory of Materials Processing and Mold (Zhengzhou University), Ministry of Education, Zhengzhou, Henan, China

*: Corresponding author: [hao.chen@griffith.edu.au](mailto:hao.chen@griffith.edu.au) (H. Chen), [s.zhang@griffith.edu.au](mailto:s.zhang@griffith.edu.au) (S. Zhang)

## Materials Characterisation

Elemental Sulfur, Carbon Black (CB), lowly-branched polysaccharide (LBP), Poly(vinylidene fluoride) (PVDF), 1,3-dioxolane (DOL), 1,2-dimethoxyethane (DME), bis(trifluoromethane)sulfonimide (LiTFSI), lithium nitrate (LiNO_3_), lithium sulfide (Li_2_S), and 1-methyl-2-pyrrolidinone (NMP) were obtained from Sigma-Aldrich and used directly without any further purification. Thermogravimetric analysis (TGA) was carried out on a Netzsch STA 449 F3 Jupiter (Netzsch, Germany) at a temperature ranging from RT to 650 ^o^C at a heating rate of 15 ^o^C·min^-1^ under an argon atmosphere. X-ray diffraction (XRD) patterns were conducted in a Model LabX-6000 diffractometer (Shimadzu, Japan) using Cu Kα radiation (λ = 1.54 Å) at 40 kV and 40 mA between the 2θ range of 10 - 80 °. For the polysulfide adsorption experiment, a 0.01 M Li_2_S_6_ solution was prepared by adding Li_2_S and elemental sulfur in a 1:5 molar ratio to a solvent of DOL:DME (1:1 v/v) in an argon-filled glovebox, before being magnetically stirred at 70 ^o^C for 24 h. 100 mg of PVDF, LBP, and HBA were exposed to 20 mL of 0.01 M Li_2_S_6_ solution for 4 h. Then, an aliquot of the supernatant solution was taken, and UV-Vis spectroscopy was carried out on a Cary Series UV-Vis-NIR Spectrophotometer (Agilent Technologies, USA). Fourier transform infrared (FTIR) spectroscopy was carried out on a Bruker Alpha (Bruker, USA) in absorbance mode to compare the spectra of the PVDF, LBP, and HBA samples before and after Li_2_S_6_ exposure. Scanning electron microscope (SEM) images and Energy-dispersive X-ray spectroscopy (EDS) data were obtained on a JSM-7001F SEM (JEOL, Japan) and was used to investigate the morphologies and elemental distributions of the S/PVDF, S/LBP, and S/HBA electrodes before and after cycling.

## Mechanical Characterisation

The peeling tests were carried out on the three electrodes with a coating thickness of approx. 30 µm at a constant speed of 1 mm·s^-1^ on an MTS Tytron microforce tester (MTS, USA). The peeling test samples were fixed on aluminium substrates, and the electrode materials were peeled off from current collectors using Scotch Sticky Tape (12 mm in width). The nano-indentation, nano-scratching, and morphology mapping were performed using Hysitron TI 950 nano-indentation system (Hysitron, USA). The reduced modulus and hardness were obtained by the nano-indentation with a Berkovich indenter. The force for nano-indentation was kept at 2000 µN, and the holding time was set to 10 s. The conical indenter with a tip 1 µm in diameter was used to perform scratching on the surface of the electrodes and the morphology mapping after scratching.


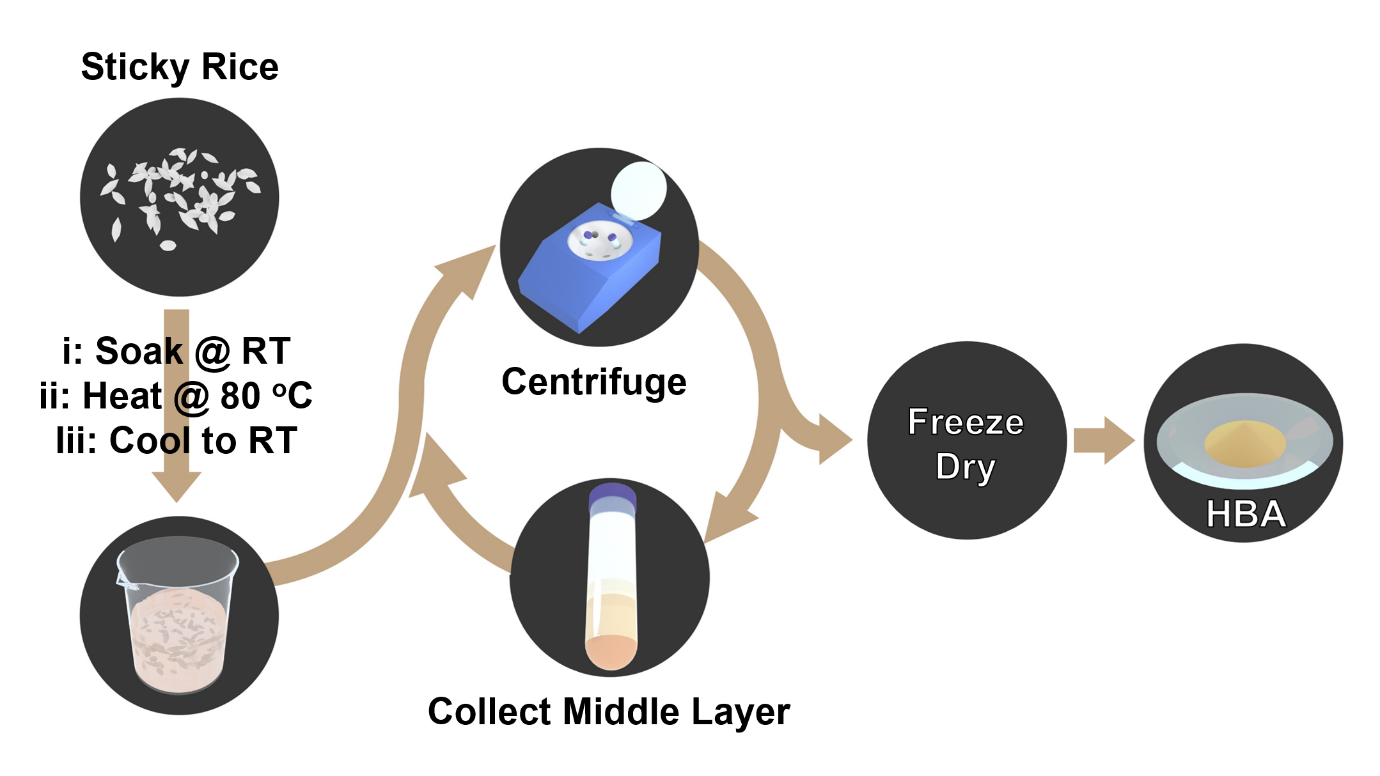


**Figure S1:** Extraction and purification of the highly-branched amylopectin (HBA) binder from sticky rice


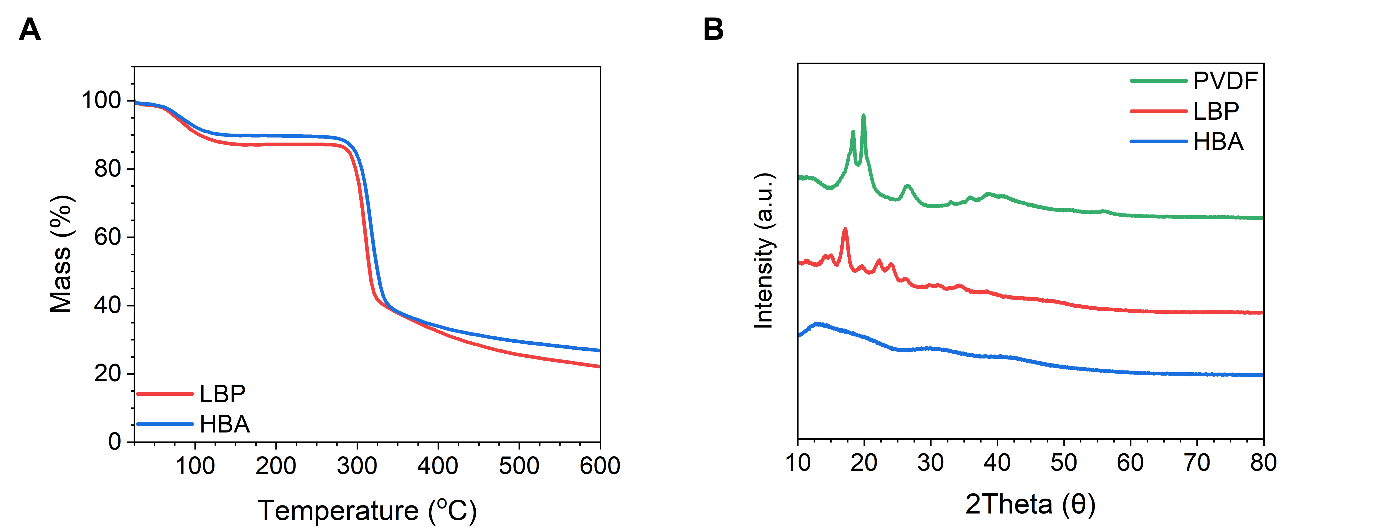


**Figure S2:** (A) Thermogravimetric analysis (TGA) results for the LBP and HBA binders. (B) X-ray diffraction (XRD) patterns for the PVDF, LBP, and HBA binders


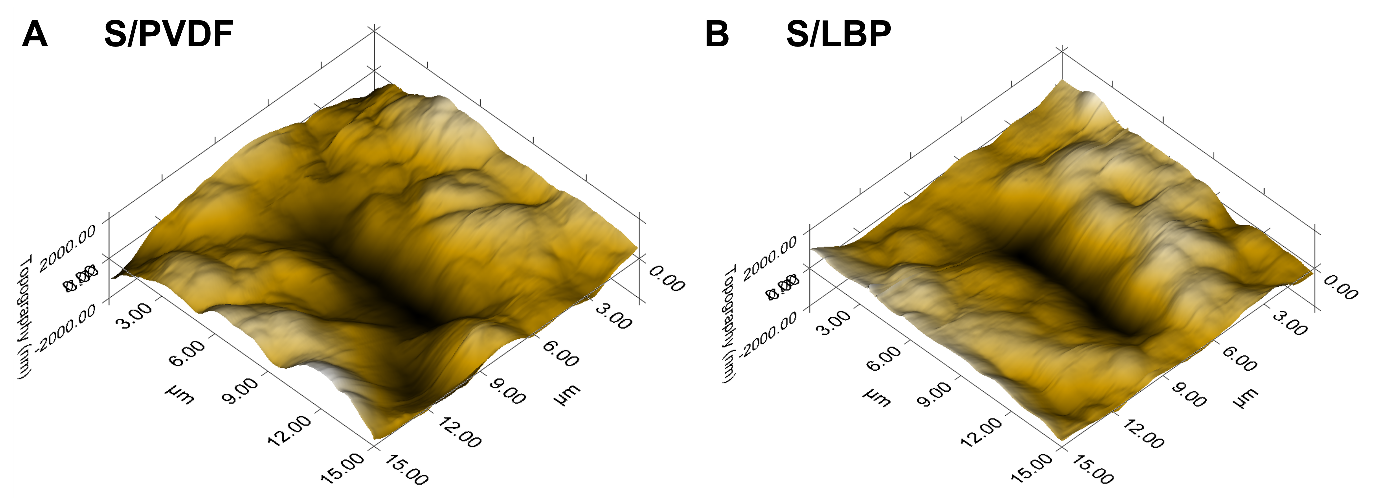


**Figure S3:** SPM images from the nano-scratch test for the (A) S/PVDF and (B) S/LBP electrodes


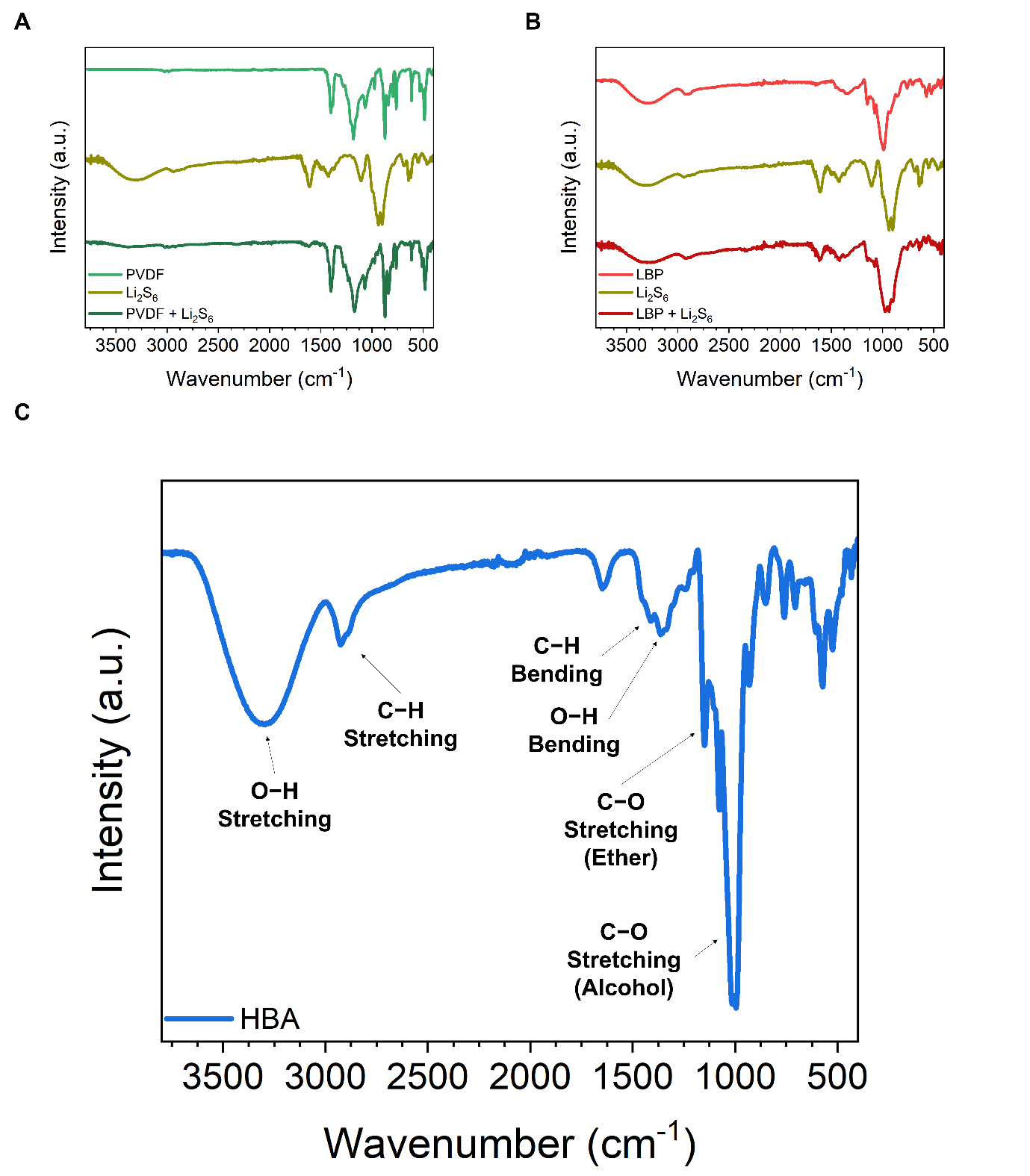


**Figure S4:** FTIR spectra of the (A) PVDF, Li_2_S_6_, and PVDF + Li_2_S_6_ samples, (B) LBP, Li_2_S_6_, and LBP + Li_2_S_6_ samples, and (C) Peak assigned FTIR spectra of the HBA binder


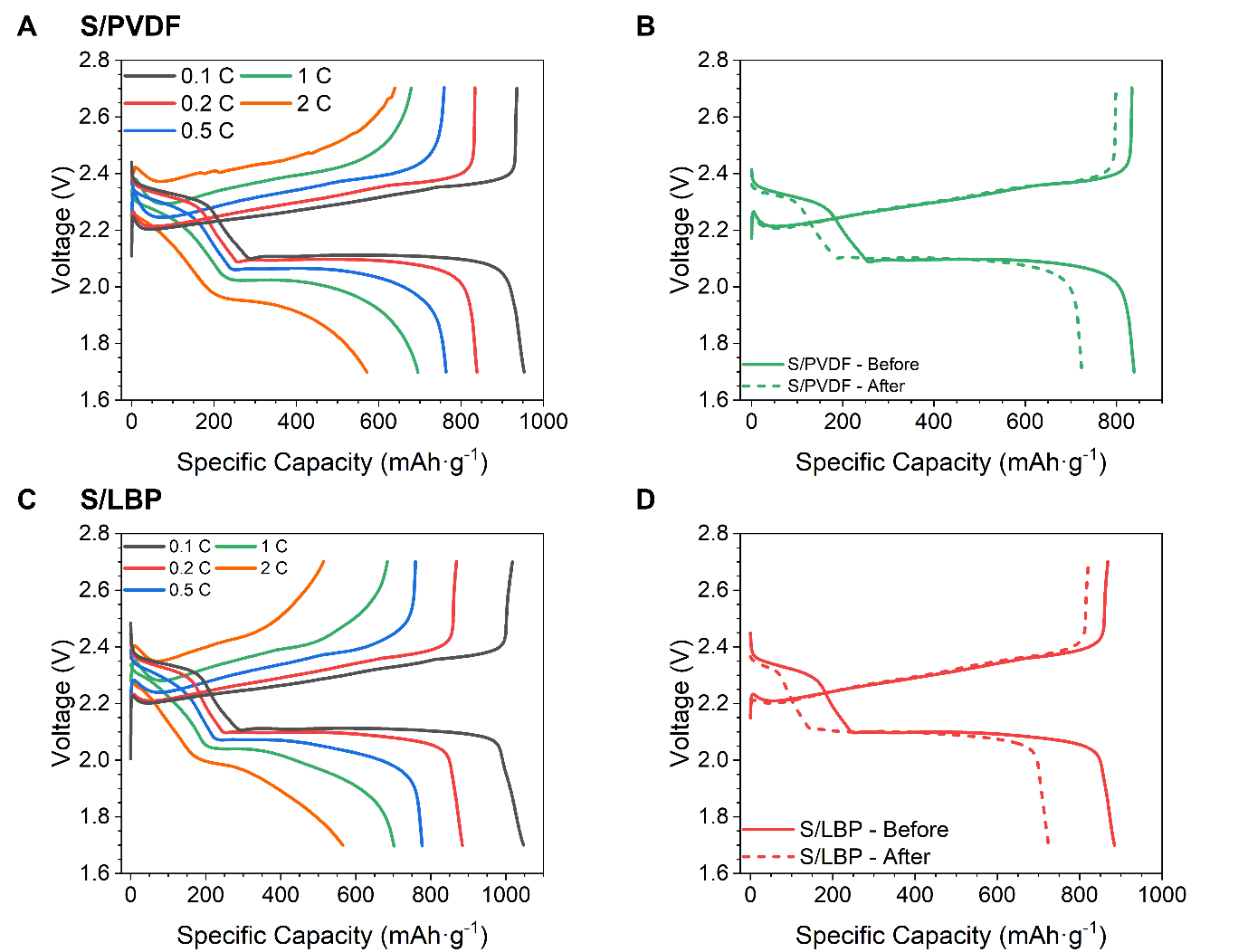


**Figure S5:** Capacity/Voltage profiles of the (A, B) S/PVDF and (C, D) S/LBP cells during rate capability testing and for the two cycles at 0.2 C during and after rate capability testing


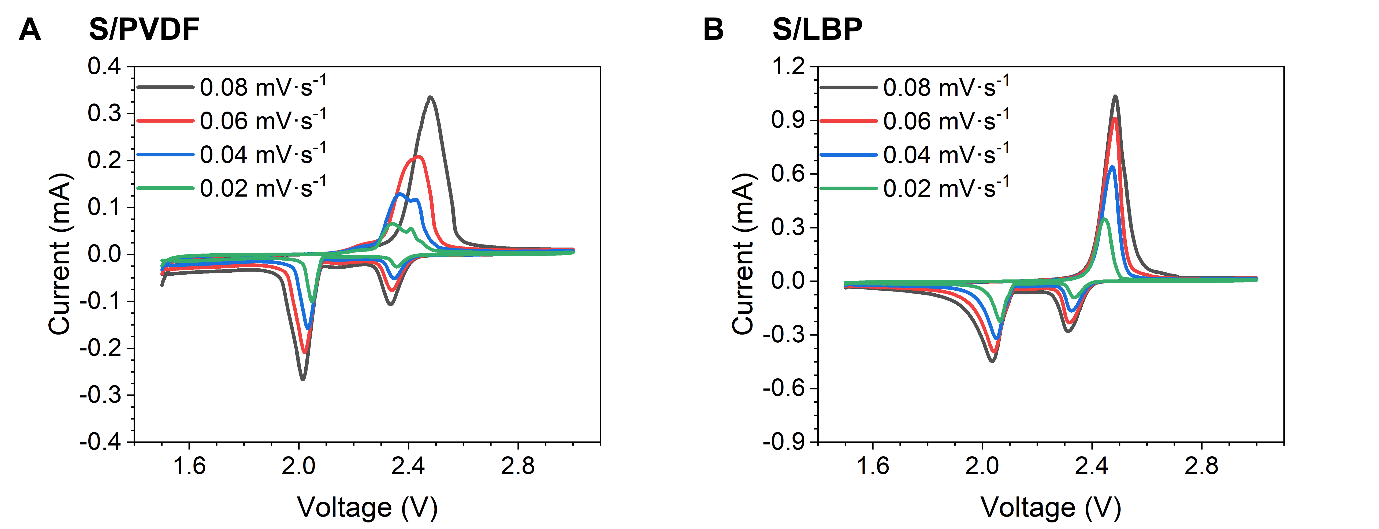


**Figure S6:** Cyclic Voltammograms (CV) from the (A) S/PVDF and (B) S/LBP electrodes


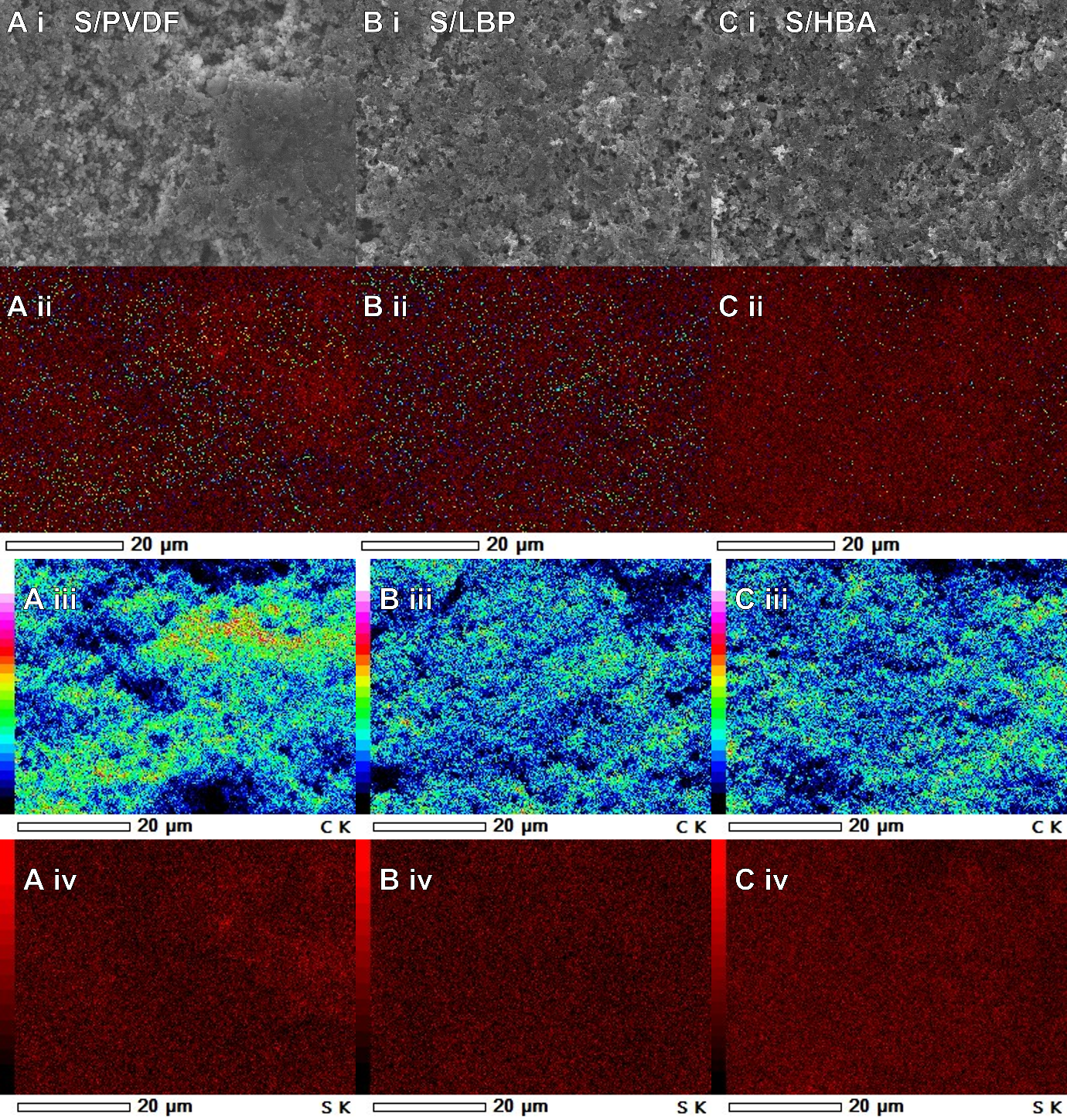


**Figure S7:** Energy-dispersive X-ray spectroscopy (EDS) results showing the (i) SEM image, (ii) elemental mapping overlay, (iii) carbon distribution, and (iv) sulfur distribution for the (A) S/PVDF, (B) S/LBP, and (C) S/HBA electrode after cycling
